# Medication Adherence, Treatment Attitudes, and Beliefs About Medicines in Romanian Psychiatric Patients: A Cross-Sectional Study

**DOI:** 10.3390/diseases14060222

**Published:** 2026-06-21

**Authors:** Antonia Ioana Vasile, Andreea Arsene, Ioana Raluca Petru

**Affiliations:** 1 Doctoral School, “Carol Davila” University of Medicine and Pharmacy, 041914 Bucharest, Romania; 2 Health Psychology Department, Faculty of Psychology and Educational Sciences, University of Bucharest, 050663 Bucharest, Romania; andreea.arsene81@s.fpse.unibuc.ro; 3 Psychology Office Private Practice Department, 060074 Bucharest, Romania; petruralucapsi@gmail.com

**Keywords:** medication adherence, psychiatric medication, treatment attitudes, beliefs about medicines, psychopharmacological treatment

## Abstract

Background: Medication adherence is a major determinant of treatment effectiveness in psychiatric care and is influenced by patients’ attitudes toward medication and beliefs about treatment. Objective: This study aimed to evaluate medication adherence, drug attitudes, and beliefs about medicines, and to examine their relationships in the study population. Methods: A total of 300 participants were assessed using the Medication Adherence Rating Scale (MARS), Drug Attitude Inventory-10 (DAI-10), and Beliefs about Medicines Questionnaire (BMQ-General and BMQ-Specific). Descriptive statistics, independent-samples *t*-tests, Pearson correlation analyses, and multiple linear regression were performed. Results: The mean DAI-10 score was 3.57 ± 3.44, indicating an overall positive attitude toward medication, although 27.33% of participants had neutral or negative attitudes. The mean MARS score was 6.27 ± 2.24, suggesting moderate adherence. Mean BMQ-General and BMQ-Specific scores were 21.70 ± 5.81 and 31.64 ± 6.13, respectively. Significant gender differences were found across all scales. DAI-10 was positively correlated with MARS, while BMQ-General was negatively correlated with MARS. Multiple regression showed that DAI-10, BMQ-General, and BMQ-Specific significantly predicted MARS scores, explained 30.8% of variance after adjustment. Conclusions: Medication adherence was moderate and was significantly associated with treatment attitudes and beliefs about medicines. The findings support multidimensional assessment and targeted interventions addressing both positive attitudes and negative medication beliefs.

## 1. Introduction

Medication adherence represents a major determinant of treatment effectiveness in psychiatric care. Pharmacological treatment plays an essential role in symptom control, relapse prevention, functional recovery, and long-term prognosis; however, partial or poor adherence remains a frequent problem in psychiatric populations. Non-adherence to psychotropic medication has been associated with symptom exacerbation, relapse, rehospitalization, and poorer clinical outcomes. For example, studies on schizophrenia and severe mental illness have shown that non-adherence is a major predictor of relapse, and the recent literature continues to emphasize that more than half of patients prescribed antipsychotic medication may present some degree of non-adherence [1,2,3].

Medication adherence is a complex and multidimensional behavior influenced by clinical, psychological, social, and treatment-related factors. Treatment-related factors are important, as atypical antipsychotics differ in receptor profiles, clinical effects, tolerability, and adverse-reaction patterns, which may influence patients’ subjective experience of medication and, consequently, their adherence behavior [4]. Recent Romanian research also supports the need for a broader, multidimensional assessment in psychiatric care, showing that in major depressive disorder, cognitive dysfunction, apathy, fatigue, and symptom severity are interrelated and should be integrated into diagnostic assessment and treatment planning, rather than focusing exclusively on affective symptoms [5]. Beyond medication availability and medical recommendation, patients’ subjective perceptions of treatment strongly influence whether medication is accepted and taken consistently. Psychological variables such as attitudes toward treatment, perceived need for medication, concerns about adverse effects, insight, self-efficacy, previous treatment experiences, and general health beliefs may all contribute to adherence behavior. A systematic review of psychological factors involved in psychopharmacological adherence concluded that adherence in mental health patients is associated with health beliefs and psychological variables, including self-efficacy and locus of control, supporting the view that adherence should be understood as a multicausal phenomenon [6].

The Medication Adherence Rating Scale is one of the instruments developed specifically to assess adherence in patients with psychosis. Thompson, Kulkarni, and Sergejew introduced the MARS as a 10-item self-report scale designed to evaluate adherence behavior and attitudes toward psychoactive medication, and reported evidence for its reliability and validity in psychotic disorders [7]. Later validation works also described the MARS as a self-report measure of adherence in psychosis and emphasized that adherence is an important predictor of illness course and outcome [8]. Therefore, the MARS is clinically useful because it allows researchers to quantify adherence behavior in a standardized way and to examine its association with psychological predictors.

Attitudes toward medication are another important dimension of adherence. The Drug Attitude Inventory, including its shorter DAI-10 version, is commonly used to evaluate patients’ subjective attitudes toward psychiatric medication. Higher DAI-10 scores indicate a more favorable attitude toward medication, while lower or negative scores suggest ambivalence or negative attitudes. Previous research has shown that DAI-10 performs similarly to the longer DAI-30 and that both versions are closely correlated, supporting the use of DAI-10 as a practical measure of treatment attitude [9]. Recent studies also support the relationship between medication attitudes and adherence. For example, Hsieh et al. reported a significant association between medication attitude and adherence in community-dwelling patients with schizophrenia, while Choi and Kweon found that drug attitude was positively associated with medication adherence in patients with early psychosis [6,10,11].

Beliefs about medicines provide an additional framework for understanding adherence. The Beliefs about Medicines Questionnaire was developed by Horne, Weinman, and Hankins to assess patients’ cognitive representations of medication, including both general beliefs about medicines and beliefs about prescribed medication [12]. The BMQ is particularly relevant because patients may simultaneously recognize the necessity of treatment while also having concerns about dependence, adverse effects, harm, or overuse of medicines. The Necessity–Concerns Framework proposes that adherence is influenced by the balance between perceived personal need for medication and concerns about potential negative consequences. A meta-analytic review of this framework showed that stronger necessity beliefs are generally associated with better adherence, whereas stronger concerns are associated with poorer adherence across illness groups [13].

In psychiatric populations, the BMQ has also been shown to be relevant for adherence. Jónsdóttir et al. examined beliefs about medication in patients with severe mental disorders and concluded that the BMQ has satisfactory psychometric properties in this population and that the beliefs measured by the questionnaire are related to adherence [14]. We consider this important, because psychiatric patients may have specific concerns related to psychotropic treatment, including fear of side effects, stigma, dependence, long-term harm, or doubts about treatment necessity. Recent studies further support the clinical importance of medication beliefs; for example, research in schizophrenia has shown that higher specific concern scores and side-effect burden are associated with lower odds of adherence [15].

Although previous studies have examined medication adherence, drug attitudes, and medication beliefs in psychiatric populations, relatively few have integrated these constructs within the same analytical model. De las Cuevas et al. showed that adherence in psychiatric outpatients was significantly associated with DAI-10 scores and BMQ necessity and concern beliefs, while a large cross-cultural study found that pharmacophobia and skepticism toward medication were consistently related to non-adherence [16,17]. More recently, Yavuz et al. further demonstrated the relevance of jointly assessing DAI-10 and BMQ dimensions in stable psychiatric patients [18]. However, the present study extends this literature by analyzing DAI-10, BMQ-General, BMQ-Specific, and MARS total scores together in the same population and by testing their combined predictive contribution to medication adherence. Examining these variables simultaneously is important because adherence is unlikely to be explained by a single factor. A patient may have a generally positive attitude toward treatment but still show incomplete adherence due to concerns about harm, low perceived necessity, side effects, stigma, or broader negative beliefs about medicines. Therefore, integrating adherence behavior, treatment attitude, and medication beliefs may provide a more complete understanding of the psychological mechanisms associated with adherence.

The aim of the present study was to evaluate medication adherence, attitudes toward medication, and beliefs about medicines in the study population using the MARS, DAI-10, BMQ-General, and BMQ-Specific scales. The study also aimed to examine gender differences in these variables, analyze the correlations between adherence, attitudes, and beliefs, and determine whether DAI-10, BMQ-General, and BMQ-Specific scores predict medication adherence measured by MARS. By combining these instruments, the study seeks to contribute to a more integrated understanding of adherence and to identify psychological factors that may be useful targets for psychoeducational and adherence-focused interventions.

Based on the theoretical and empirical literature reviewed above, we formulated the following hypotheses. First, higher DAI-10 scores, reflecting more positive attitudes toward psychiatric medication, were expected to be associated with higher MARS scores, indicating better medication adherence. Second, higher BMQ-General scores, reflecting stronger general concerns or negative beliefs about medicines, were expected to be associated with lower MARS scores. Third, BMQ-Specific scores were expected to be significantly associated with MARS scores, indicating that patients’ beliefs about their own prescribed medication would be related to adherence behavior. Finally, DAI-10, BMQ-General, and BMQ-Specific scores were expected to jointly predict MARS scores in the multiple regression model.

## 2. Materials and Methods

The research design adopted in this study is quantitative, cross-sectional, and non-experimental, employing a post-positivist approach specific to applied research. The study focused on exploring the relationships between measurable psychological variables, without direct intervention by the researcher, in a naturalistic clinical setting, where patients were already undergoing psychiatric treatment.

The research aimed to identify patients’ attitudes toward psychiatric medication and how these attitudes influence treatment adherence. Given the proposed objectives, a descriptive-correlational strategy was used, which allows for the examination of statistical associations between distinct psychological variables without establishing causality.

Medication adherence was assessed using the Medication Adherence Rating Scale-10 (MARS-10). The MARS-10 includes 10 dichotomous items and yields a total score ranging from 0 to 10, with higher scores indicating better medication adherence. According to the scoring procedure, items 1–6 and 9–10 were scored as adherent when the response was “No/0”, whereas items 7–8 were scored as adherent when the response was “Yes/1”. For descriptive purposes, MARS-10 scores were categorized as low adherence (0–4 points), moderate adherence (5–7 points), and high adherence (8–10 points). These categories were used only to describe the distribution of adherence levels in the sample.

Attitudes toward psychiatric medication were assessed using the Drug Attitude Inventory-10 (DAI-10). The DAI-10 includes 10 items scored as +1 or −1 according to the standard scoring key, producing a total score ranging from −10 to +10. Higher scores indicate more positive attitudes toward medication. For descriptive classification, scores below 0 were classified as negative attitude, a score of exactly 0 was classified as neutral attitude, and scores above 0 were classified as positive attitude.

Beliefs about medicines were assessed using the Beliefs about Medicines Questionnaire (BMQ). The BMQ-General includes 8 items rated on a 5-point Likert scale and yields a total score ranging from 8 to 40, with higher scores indicating stronger general beliefs or concerns about medicines as a class. The BMQ-Specific includes 10 items rated on a 5-point Likert scale and yields a total score ranging from 10 to 50, with higher scores indicating stronger beliefs about the participant’s own prescribed medication. In the present study, BMQ-General and BMQ-Specific total scores were used in the main analyses.

Data collection was conducted at a single point in time (cross-sectional design) through the individual, self-administered application of a set of validated psychometric questionnaires: DAI-10 (Drug Attitude Inventory) to assess overall attitude toward medication; MARS (Medication Adherence Rating Scale) to estimate adherence behavior; BMQ (Beliefs about Medicines Questionnaire, in its general and specific components) to investigate cognitive representations related to treatment. In the current Romanian sample, internal consistency varied across the instruments. The MARS-10 showed borderline acceptable reliability, with Cronbach’s alpha = 0.70. The BMQ-General showed moderate/questionable internal consistency, with Cronbach’s alpha = 0.62. Lower internal consistency was observed for the DAI-10, Cronbach’s alpha = 0.36, and for the BMQ-Specific, Cronbach’s alpha = 0.49. These values indicate that results involving DAI-10 and BMQ-Specific should be interpreted with caution, while also considering that these instruments assess heterogeneous treatment-related attitudes and beliefs. For the BMQ, a strong improvement would have been to report reliability separately for BMQ-General Harm, BMQ-General Overuse, BMQ-Specific Necessity, and BMQ-Specific Concerns, rather than only BMQ-General and BMQ-Specific totals.

Participants were recruited from a psychiatric medical setting between November 2024 and December 2025 and were evenly divided into four approximately equal categories: women diagnosed with depression, women diagnosed with schizophrenia, men diagnosed with depression, and men diagnosed with schizophrenia. This grouping allowed for the examination of differences in attitude and adherence based on gender and diagnosis. A non-probability consecutive sampling strategy was used. Specifically, all patients attending or hospitalized in the participating psychiatric clinical setting during the recruitment period were screened for eligibility in the order in which they became available to the research team. Patients who met the inclusion criteria and did not meet any exclusion criteria were invited to participate until the planned balanced structure by gender and diagnosis was achieved. Therefore, recruitment was sequential and clinically based, rather than randomized. This approach was chosen because the study was conducted in a hospital psychiatric setting and aimed to include adult patients receiving active psychiatric medication treatment for schizophrenia or major depressive episode. All participants signed an informed consent form after being clearly and thoroughly informed in advance about the purpose of the study, the estimated time required to complete the questionnaires, data confidentiality, and their right to withdraw at any time without providing a reason. The questionnaires were completed anonymously, and no personal data was collected during the study. No monetary rewards or other forms of compensation were offered; participation was entirely voluntary under the supervision of a psychiatrist and a researcher. The study was conducted in accordance with the Declaration of Helsinki, and approved by the Institutional Review Board of FPSE, University of Bucharest.

In the analysis phase, descriptive statistical methods (means, standard deviations) and inferential methods were applied, such as *t*-tests for independent samples, to compare scores by gender; Pearson correlation coefficients, to examine the relationships between scale scores; multiple linear regression analysis, to identify the strongest predictors of adherence (MARS).

Before conducting parametric analyses, the assumptions underlying independent-samples *t*-tests, Pearson correlations, and multiple linear regression were examined. Because the main variables were total scores derived from multi-item self-report scales, DAI-10, MARS-10, BMQ-General, and BMQ-Specific scores were treated as continuous variables. Normality was assessed using Shapiro–Wilk tests, skewness and kurtosis values, histograms, and Q–Q plots. Homogeneity of variance for gender comparisons was evaluated using Levene’s test; when this assumption was not met, Welch’s correction was applied. Linearity between variables was assessed by inspecting scatterplots. For the multiple regression model, residual plots were examined to evaluate linearity and homoscedasticity, Q–Q plots and the Shapiro–Wilk test were used to assess residual normality, and multicollinearity was evaluated using tolerance and variance inflation factor values. Influential observations were examined using standardized residuals and Cook’s distance. Parametric analyses were retained because the assumption diagnostics supported their use, and because the sample size was sufficiently large for the analyses to be robust to minor deviations from normality.

The initial screened sample consisted of 398 Romanian patients recruited from a psychiatric clinical setting at the Clinical Psychiatric Hospital in Bucharest. Of these, 98 patients were excluded before analysis: 48 did not meet the selection criteria, 26 had additional major psychiatric comorbidities, 20 had severe cognitive, language, intellectual, or other difficulties that could have affected response accuracy, and 4 refused to participate or withdrew before completing the questionnaires. Therefore, the final analyzed sample consisted of 300 participants.

Eligibility criteria required participants to be adults over 18 years of age, currently undergoing active psychiatric medication treatment, and to have a confirmed clinical diagnosis of schizophrenia or major depressive episode based on medical evaluations in their clinical records. Participants were also required to understand and independently complete the study questionnaires.

The final analyzed sample included 150 women and 150 men. By diagnosis, 160 participants had major depression, including 80 women and 80 men, and 140 participants had schizophrenia, including 70 women and 70 men.

Assuming a small to medium effect size of f^2^ = 0.08, an alpha level of 0.05, and desired statistical power of 0.80, the analysis indicated a minimum required sample size of 166 participants for detecting the effect of the predictors on the outcome within the regression framework.

**Figure 1:**
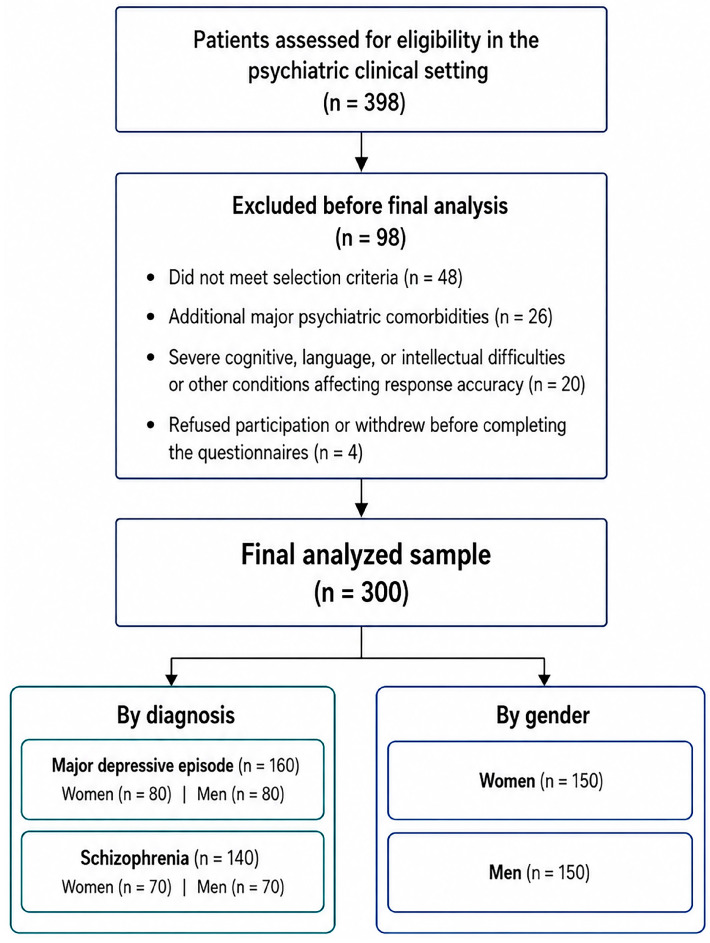
Participant flow diagram. Derivation of the final analyzed sample.

Demographically, the sample was intended to be balanced by gender and diagnosis: 150 women and 150 men, of whom 80 women and 80 men had a diagnosis of major depression, and 70 women and 70 men had a diagnosis of schizophrenia (Figure 1). Age was not explicitly collected, but all participants fell within the adult age range (over 18 years old) and were considered clinically active. This balanced sample structure was essential to enable a rigorous comparative analysis of differences in attitudes toward psychiatric medication and levels of treatment adherence based on gender and diagnosis.

## 3. Results

To assess patients’ attitudes toward drug therapy, the scores obtained on the four instruments used—the DAI-10, MARS, BMQ-General, and BMQ-Specific—were analyzed. To provide a clearer descriptive overview of clinically relevant response patterns, participants were also classified into adherence and attitude categories. MARS-10 scores were grouped into low adherence, moderate adherence, and high adherence categories, while DAI-10 scores were grouped into negative, neutral, and positive attitude categories. The distribution of participants across these categories is presented in Table 1.

The mean DAI-10 score was 3.57 ± 3.44, indicating an overall positive attitude toward medication, although with substantial interindividual variability. Based on the predefined descriptive categories, 22 participants (7.33%) had a negative attitude, 60 participants (20.00%) had a neutral attitude, and 218 participants (72.67%) had a positive attitude toward medication.

The mean MARS-10 score was 6.27 ± 2.24, suggesting a moderate overall level of medication adherence in the study population. Based on the predefined descriptive categories, 76 participants (25.33%) showed low adherence, 105 participants (35.00%) showed moderate adherence, and 119 participants (39.67%) showed high adherence.

The mean BMQ-General score was 21.70 ± 5.81, suggesting moderate general beliefs or concerns about medicines as a class. The mean BMQ-Specific score was 31.64 ± 6.13, indicating moderately strong beliefs about participants’ own prescribed medication.

**Figure 2:**
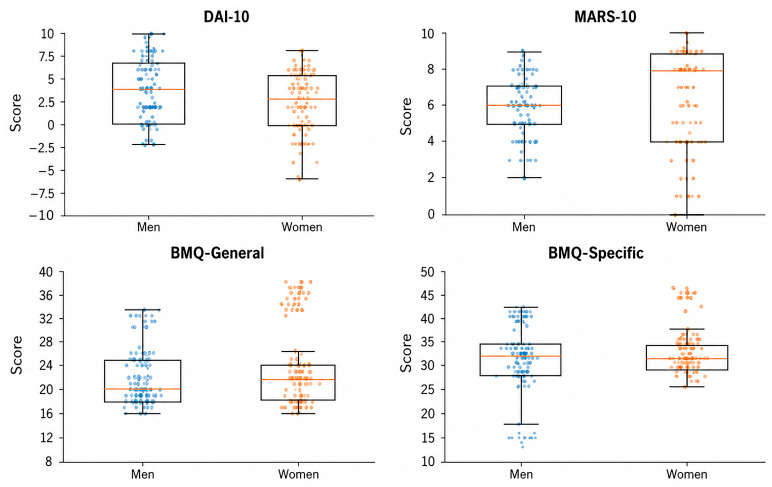
Distribution of DAI-10, MARS-10, BMQ-General, and BMQ-Specific scores by gender. Box plots show the median and interquartile range, with individual participant scores overlaid as jittered points. Men and women represent independent groups.

The distribution of DAI-10, MARS-10, BMQ-General, and BMQ-Specific scores by gender is shown in Figure 2. The plots display individual participant scores overlaid on box plots, allowing visual inspection of group differences, score dispersion, potential outliers, and the bounded distribution of the scales.

Independent-samples Welch *t*-tests showed statistically significant gender differences across all analyzed scales. Men obtained significantly higher DAI-10 scores than women, indicating more positive attitudes toward medication: t(279.239) = 3.200, *p* = 0.0015, Cohen’s d = 0.370, 95% CI [0.141, 0.598]. Women obtained significantly higher MARS-10 scores than men, indicating better medication adherence: t(260.190) = −2.653, *p* = 0.0085, Cohen’s d = −0.306, 95% CI [−0.534, −0.078]. Women also scored significantly higher on BMQ-General: t(258.473) = −3.518, *p* = 0.0005, Cohen’s d = −0.406, 95% CI [−0.635, −0.177]; and BMQ-Specific: t(258.247) = −2.589, *p* = 0.0102, Cohen’s d = −0.299, 95% CI [−0.526, −0.071]. Although all gender differences were statistically significant, the effect sizes were small to small-to-moderate, suggesting that the magnitude of these differences should be interpreted cautiously in terms of clinical relevance (Table 2).

Independent-samples *t*-tests showed significant gender differences across all analyzed scales. Male participants obtained significantly higher DAI-10 scores than female participants, indicating a more positive attitude toward medication. In contrast, female participants showed significantly higher MARS-10 scores, suggesting better medication adherence. Female participants also scored significantly higher on both the BMQ-General and BMQ-Specific scales, indicating stronger general beliefs or concerns about medicines and stronger beliefs regarding their prescribed medication.

Because all four scale scores differed significantly by gender, gender was added to the regression analysis as a covariate. Female gender was coded as 1 and male gender as 0. The gender-adjusted model was statistically significant: R^2^ = 0.400, adjusted R^2^ = 0.392, F(4, 295) = 49.19, *p* < 0.001. DAI-10 remained a significant positive predictor of MARS-10 scores: B = 0.273, 95% CI [0.212, 0.333], *p* < 0.001; and BMQ-General remained a significant negative predictor: B = −0.165, 95% CI [−0.206, −0.124], *p* < 0.001. BMQ-Specific was not significant in the gender-adjusted model: B = 0.031, 95% CI [−0.007, 0.069], *p* = 0.112. Gender was independently associated with adherence, with female participants showing higher MARS-10 scores after adjustment for DAI-10, BMQ-General, and BMQ-Specific: B = 1.349, 95% CI [0.938, 1.760], *p* < 0.001.

Gender-stratified regressions were then conducted. Among men, the model explained 12.5% of the variance in MARS-10 scores: R^2^ = 0.125, adjusted R^2^ = 0.107, F(3, 146) = 6.96, *p* < 0.001. In this group, DAI-10 was a significant positive predictor: B = 0.086, 95% CI [0.010, 0.162], *p* = 0.027; and BMQ-Specific was also a significant positive predictor: B = 0.060, 95% CI [0.016, 0.103], *p* = 0.008. BMQ-General was not significant among men: B = −0.004, 95% CI [−0.071, 0.064], *p* = 0.918. Among women, the model explained 67.5% of the variance in MARS-10 scores: R^2^ = 0.675, adjusted R^2^ = 0.668, F(3, 146) = 100.86, *p* < 0.001. In this group, DAI-10 was a significant positive predictor: B = 0.434, 95% CI [0.327, 0.541], *p* < 0.001, and BMQ-General was a significant negative predictor: B = −0.180, 95% CI [−0.228, −0.132], *p* < 0.001. BMQ-Specific was not significant among women: B = 0.013, 95% CI [−0.057, 0.083], *p* = 0.721 (Table 3).

Model fit: R^2^ = 0.400, adjusted R^2^ = 0.392, F(4, 295) = 49.19, *p* < 0.001.

This means that gender remains independently associated with MARS adherence scores even after accounting for DAI-10, BMQ-General, and BMQ-Specific.

A formal interaction model was also tested to examine whether the associations between predictors and adherence differed by gender (Table 4). The interaction block was statistically significant, F(3, 292) = 23.30, *p* < 0.001. The gender × DAI-10 interaction was significant, indicating that the association between positive medication attitude and adherence was stronger among women. The gender × BMQ-General interaction was also significant, indicating that general negative beliefs about medicines were more strongly related to adherence among women. The gender × BMQ-Specific interaction was not significant.

Model fit:

Men: R^2^ = 0.125, adjusted R^2^ = 0.107, F(3, 146) = 6.96, *p* < 0.001.

Women: R^2^ = 0.675, adjusted R^2^ = 0.668, F(3, 146) = 100.86, *p* < 0.001.

A formal interaction model also supported gender-specific patterns: the combined interaction block was significant: F(3, 292) = 23.30, *p* < 0.001. The gender × DAI-10 interaction was significant: *p* < 0.001; the gender × BMQ-General interaction was significant: *p* < 0.001; and the gender × BMQ-Specific interaction was not significant: *p* = 0.273.

Because diagnosis was a design variable, additional analyses were conducted to examine whether scale scores differed between patients with major depressive episode and patients with schizophrenia. The final analyzed sample included 160 participants with major depressive episode and 140 participants with schizophrenia. Patients with major depressive episode had significantly higher DAI-10 scores than patients with schizophrenia: 4.41 ± 3.71 versus 2.61 ± 2.83, mean difference = 1.80, 95% CI [1.05, 2.54], *p* < 0.001, Cohen’s d = 0.54. No statistically significant diagnostic difference was observed for MARS-10 scores: 6.39 ± 2.28 versus 6.12 ± 2.20, mean difference = 0.27, 95% CI [−0.24, 0.78], *p* = 0.294, Cohen’s d = 0.12. BMQ-General scores also did not differ significantly between groups: 21.30 ± 5.22 versus 22.16 ± 6.43, mean difference = −0.86, 95% CI [−2.20, 0.49], *p* = 0.210, Cohen’s d = −0.15. However, BMQ-Specific scores were significantly higher in the depression group than in the schizophrenia group: 34.56 ± 5.24 versus 28.30 ± 5.37, mean difference = 6.26, 95% CI [5.05, 7.47], *p* < 0.001, Cohen’s d = 1.18 (Table 5).

To examine whether the main regression findings remained after accounting for diagnosis, diagnosis was added as a covariate in the prediction of MARS-10 scores. The adjusted model was statistically significant: R^2^ = 0.341, adjusted R^2^ = 0.332, F(4, 295) = 38.23, *p* < 0.001. DAI-10 remained a significant positive predictor of MARS-10 scores: B = 0.265, 95% CI [0.202, 0.329], *p* < 0.001, while BMQ-General remained a significant negative predictor: B = −0.184, 95% CI [−0.230, −0.137], *p* < 0.001. BMQ-Specific was also a significant positive predictor in the diagnosis-adjusted model: B = 0.096, 95% CI [0.045, 0.147], *p* < 0.001. Diagnosis also contributed significantly to the model, with schizophrenia coded as 1 and depression as 0: B = 0.961, 95% CI [0.407, 1.516], *p* = 0.001 (Table 6).

Because the clinical meaning of adherence, beliefs, and medication attitudes may differ between major depressive episode and schizophrenia, diagnosis-stratified regressions were also conducted. In the depression group, the regression model explained 64.1% of the variance in MARS-10 scores: R^2^ = 0.641, adjusted R^2^ = 0.634, F(3, 156) = 92.99, *p* < 0.001. DAI-10 was a significant positive predictor: B = 0.161, 95% CI [0.098, 0.223], *p* < 0.001, and BMQ-General was a significant negative predictor: B = −0.335, 95% CI [−0.398, −0.272], *p* < 0.001. BMQ-Specific was not a significant predictor in this group: B = 0.042, 95% CI [−0.020, 0.104], *p* = 0.187 (Table 7).

In the schizophrenia group, the regression model explained 63.9% of the variance in MARS-10 scores: R^2^ = 0.639, adjusted R^2^ = 0.631, F(3, 136) = 80.25, *p* < 0.001. DAI-10 was a significant positive predictor: B = 0.652, 95% CI [0.561, 0.743], *p* < 0.001, BMQ-General was a significant negative predictor: B = −0.137, 95% CI [−0.179, −0.094], *p* < 0.001, and BMQ-Specific was a significant positive predictor: B = 0.344, 95% CI [0.287, 0.401], *p* < 0.001 (Table 8). Formal diagnosis-by-predictor interaction analyses supported diagnosis-specific patterns, indicating that the associations between medication beliefs, attitudes, and adherence should not be interpreted as identical across the two diagnostic groups.

A formal interaction model also supported diagnosis-specific patterns: diagnosis × DAI-10, diagnosis × BMQ-General, and diagnosis × BMQ-Specific interactions were all significant. It means the pooled regression should not be interpreted as one uniform mechanism across both disorders.

To explore the association between patients’ attitudes towards psychiatric medication and the level of adherence to treatment, Pearson correlation coefficients were calculated between scores obtained on the DAI-10, BMQ-General, BMQ-Specific and MARS scales (Table 9).

Pearson correlation analysis showed a significant positive correlation between DAI-10 and MARS scores, indicating that a more positive attitude toward medication was associated with better medication adherence. DAI-10 was weakly but significantly negatively correlated with BMQ-General scores. MARS scores were moderately and significantly negatively correlated with BMQ-General scores, suggesting that stronger general concerns or negative beliefs about medicines were associated with lower adherence. No significant correlations were observed between BMQ-Specific and either DAI-10 or MARS scores. BMQ-General and BMQ-Specific scores were moderately and positively correlated. After Bonferroni correction across the six correlation analyses, DAI-10 remained significantly positively correlated with MARS-10: r = 0.447, adjusted *p* < 0.001, and significantly negatively correlated with BMQ-General: r = −0.191, adjusted *p* = 0.0053. MARS-10 remained significantly negatively correlated with BMQ-General: r = −0.406, adjusted *p* < 0.001. BMQ-General and BMQ-Specific also remained significantly positively correlated: r = 0.507, adjusted *p* < 0.001. The correlations between DAI-10 and BMQ-Specific, and between MARS-10 and BMQ-Specific, were not statistically significant after correction.

To examine the extent to which patients’ attitudes towards psychiatric medication predict adherence, a multiple linear regression analysis was performed. The criterion variable was the total score on the MARS (Medication Adherence Rating Scale), and the predictor variables were the total scores on the DAI-10, BMQ-General, and BMQ-Specific (Table 10 and Table 11).

The model was statistically significant and explained approximately 30.8% of variance after adjustment in MARS adherence scores.

A multiple linear regression analysis was conducted to examine whether DAI-10, BMQ-General, and BMQ-Specific scores predicted medication adherence measured by MARS. The model was statistically significant: F(3, 296) = 45.459, *p* < 0.001, explaining 30.8% of variance after adjustment in MARS scores. DAI-10 was a significant positive predictor, while BMQ-General was a significant negative predictor of MARS scores. BMQ-Specific showed a borderline positive association with MARS scores.

Regression diagnostics were examined to assess the validity of the multiple linear regression model. Multicollinearity was not problematic, as tolerance values ranged from 0.703 to 0.946 and VIF values ranged from 1.057 to 1.422. Specifically, tolerance/VIF values were 0.946/1.057 for DAI-10, 0.703/1.422 for BMQ-General, and 0.730/1.370 for BMQ-Specific. Residual analysis showed no severe outliers, with a maximum standardized residual of 2.20, and no influential cases, with a maximum Cook’s distance of 0.030. The Durbin–Watson statistic was 2.099, indicating no evidence of residual autocorrelation. However, the Shapiro–Wilk test indicated a statistically significant departure from residual normality (W = 0.947, *p* < 0.001), and the Breusch–Pagan test indicated heteroscedasticity (LM = 14.771, *p* = 0.002). Therefore, the regression findings should be interpreted with some caution. As a sensitivity check, the model was also examined using HC3 robust standard errors; DAI-10 and BMQ-General remained significant predictors of MARS scores, whereas BMQ-Specific remained borderline and did not reach conventional statistical significance: *p* = 0.059 (Table 12).

The results indicate that the study population generally showed a moderately positive attitude toward medication. The mean DAI-10 score was 3.57 ± 3.44, which is above the neutral point of the scale and therefore suggests that, overall, participants tended to perceive psychiatric medication in a favorable way. However, the relatively large standard deviation shows that attitudes were not uniform across the sample. This is also supported by the finding that 27.33% of participants had neutral or negative attitudes, meaning that more than one quarter of the population may require additional psychoeducation, motivational support, or individualized discussion regarding medication.

Medication adherence, measured using the MARS scale, was also at a moderate level, with a mean score of 6.27 ± 2.24. This suggests that adherence behavior was acceptable but not optimal. In practical terms, although many participants reported behaviors consistent with medication adherence, a meaningful proportion may still have difficulties taking medication regularly, following medical recommendations, or maintaining treatment over time. The variability in MARS scores indicates that adherence is heterogeneous and may be influenced by individual beliefs, attitudes, clinical characteristics, or treatment-related factors.

The BMQ results provide additional insight into participants’ beliefs about medication. The BMQ-General mean score of 21.70 ± 5.81 suggests moderate general beliefs or concerns about medicines as a class, while the BMQ-Specific mean score of 31.64 ± 6.13 indicates moderately strong beliefs regarding participants’ own prescribed medication. This pattern suggests that participants may recognize the importance or relevance of their prescribed treatment, but at the same time may hold broader concerns about medicines in general. Such beliefs are important because they can influence how patients evaluate the benefits and risks of treatment and, ultimately, whether they adhere to prescribed medication.

The gender comparisons showed statistically significant differences across all analyzed scales. Male participants had significantly higher DAI-10 scores, suggesting a more positive attitude toward medication compared with female participants. In contrast, female participants had higher MARS scores, indicating better medication adherence, despite having lower medication attitude scores. Female participants also scored higher on both BMQ-General and BMQ-Specific scales, suggesting stronger beliefs and concerns about medicines overall. This may indicate that women in the sample were more engaged with or more reflective about medication-related issues, which could explain why they reported better adherence even though they expressed stronger concerns.

The correlation analysis further clarifies the relationships between attitudes, beliefs, and adherence. DAI-10 scores were moderately and positively correlated with MARS scores, meaning that participants with more positive attitudes toward medication tended to report better adherence. MARS scores were moderately and negatively correlated with BMQ-General scores, suggesting that stronger general concerns or negative beliefs about medicines were associated with poorer adherence. BMQ-Specific scores were not significantly correlated with either DAI-10 or MARS, indicating that general beliefs about medicines may have been more relevant to adherence behavior in this sample than beliefs about prescribed medication specifically. The positive correlation between BMQ-General and BMQ-Specific suggests that participants with stronger general beliefs about medicines also tended to report stronger beliefs about their own medication.

The multiple linear regression analysis showed that DAI-10, BMQ-General, and BMQ-Specific scores jointly predicted medication adherence, explained 30.8% of variance after adjustment in MARS scores. DAI-10 was a significant positive predictor, confirming that a more favorable attitude toward medication was independently associated with better adherence. BMQ-General was a significant negative predictor, indicating that stronger general concerns or negative beliefs about medicines were independently associated with lower adherence. BMQ-Specific had only a borderline positive effect, suggesting a weaker role once general medication beliefs and attitude toward treatment were taken into account. Overall, these findings suggest that improving medication adherence may require not only strengthening positive attitudes toward treatment, but also directly addressing general concerns and negative beliefs about medicines. The relevance of psychological factors in clinical expression and treatment behavior is also supported by psychosomatic research, which emphasizes that psychical tension and emotional distress may contribute to somatic and behavioral manifestations; this perspective reinforces the need to interpret medication adherence not only as a behavioral outcome, but also as part of a broader psychological and psychosomatic context [19].

## 4. Discussion

The present results show a profile of moderate medication adherence and generally favorable treatment-related attitudes. In this sample, the mean MARS score was 6.27 ± 2.24, indicating moderate adherence, while the mean DAI-10 score was 3.57 ± 3.44, suggesting an overall positive attitude toward medication. These values are consistent with previous psychiatric studies showing that adherence is often partial rather than optimal, even when patients report relatively positive attitudes toward treatment [7,8,20,21]. The MARS was originally developed as a 10-item self-report measure for psychosis, derived partly from the Drug Attitude Inventory, with scores ranging from low to high likelihood of adherence; therefore, the present mean above the midpoint supports the interpretation of moderate adherence rather than poor adherence [7].

The observed MARS score is very close to that reported by Bahrini et al., who found a mean total MARS score of 6.28 ± 2.37 in a psychiatric sample and interpreted adherence as “sufficiently good”. This similarity is notable because the present sample showed an almost identical mean score, although with slightly lower variability. Thus, compared with this study, the present population appears to have a very similar level of self-reported adherence [20]. By contrast, Ali et al. reported lower adherence in schizophrenia outpatients, with an MARS score of 4.63 ± 1.91, despite a high DAI-10 score of 8.02 ± 2.12. Compared with that study, the present sample had better MARS adherence but a less strongly positive DAI-10 attitude, suggesting that positive attitudes alone may not always correspond directly to higher adherence across different samples or cultural contexts [22].

The positive association observed in the present study between DAI-10 and MARS scores is supported by previous research showing that more favorable attitudes toward medication are associated with better adherence. In the present sample, DAI-10 was moderately and positively correlated with MARS scores (r = 0.447, *p* < 0.001), indicating that participants with more positive attitudes toward medication reported better adherence. Similar findings were reported by Choi and Kweon [11], who found a significant positive correlation between drug attitude and medication adherence in patients with early psychosis (r = 0.393, *p* < 0.001). Likewise, Hsieh et al. reported a strong positive association between medication attitude and adherence among community-dwelling patients with schizophrenia (r = 0.61, *p* < 0.001) [23]. A recent cross-sectional study by Kumari et al. also found a moderate positive correlation between drug attitude and medication adherence in psychiatric patients (r = 0.408) [24]. These findings are further supported by the systematic review and meta-analysis by Richardson et al., which concluded that medication attitudes are positively associated with adherence behavior in patients with psychosis [25]. Overall, the present results are consistent with previous evidence suggesting that positive attitudes toward medication represent an important psychological factor associated with better treatment adherence. In the current analysis, DAI-10 was positively correlated with MARS and remained a significant positive predictor in the regression model, indicating that more favorable medication attitudes were associated with better adherence. This agrees with evidence summarized in previous reviews and comparative studies, where negative attitudes toward treatment were repeatedly associated with non-adherence, while positive treatment attitudes were linked to better adherence. For example, the review table by Chakrabarti summarized multiple studies reporting that DAI-10 or other attitude measures were associated with adherence, including findings that positive attitudes were more common among adherent patients and that DAI-10 scores were significantly associated with adherence over follow-up [26].

The BMQ results also align with the broader literature on medication beliefs. In the present study, BMQ-General was negatively correlated with MARS and was a significant negative predictor in the regression model. This suggests that stronger general negative beliefs or concerns about medicines were associated with poorer adherence. This finding is highly consistent with Jónsdóttir et al., who studied patients with severe mental disorders and found that BMQ constructs were related to adherence; specifically, non-adherent patients felt that medication was less necessary and were more concerned about it than adherent patients. Their study also concluded that the BMQ has satisfactory psychometric properties in severe mental disorders and that medication beliefs are relevant to adherence in this population [14].

The present finding that BMQ-Specific showed only a borderline or weak independent association with MARS may reflect the way the scale was analyzed. The BMQ-Specific scale is often interpreted through its two subcomponents: Specific-Necessity and Specific-Concerns, rather than as a single total score. Prior studies based on the Necessity–Concerns Framework show that adherence is usually highest when patients believe medication is necessary and have fewer concerns, while non-adherence is more likely when concerns are high and perceived necessity is low. De las Cuevas et al. similarly reported that skepticism and pharmacophobia toward psychotropic medication negatively affect adherence, and that pharmacophobia or skepticism may be important determinants of non-adherence in psychiatric outpatients [17]. Therefore, the modest effect of the total BMQ-Specific score in the present regression may be due to combining necessity and concern items into one total score, potentially masking opposite effects.

The additional diagnosis-based analyses are important because they show that the pooled findings do not fully capture the clinical heterogeneity of the sample. Although the overall regression model suggested that DAI-10, BMQ-General, and BMQ-Specific were associated with MARS-10 adherence scores, the stratified analyses indicated that the pattern of associations differed between patients with major depressive episode and those with schizophrenia. In both diagnostic groups, more positive medication attitudes were associated with better adherence, and stronger general negative beliefs about medicines were associated with lower adherence. However, BMQ-Specific was a significant predictor only in the schizophrenia group. This suggests that beliefs about one’s own prescribed medication may have different clinical relevance depending on diagnosis, treatment context, illness insight, expected duration of pharmacotherapy, and medication-related experiences.

These findings also address the possibility that some pooled associations may be partly influenced by between-disorder differences. Depression and schizophrenia differ in symptom profile, illness insight, cognitive functioning, medication class, side-effect burden, and expected treatment duration, all of which may affect both adherence behavior and beliefs about medicines. Therefore, the diagnosis-adjusted and diagnosis-stratified analyses strengthen the interpretation of the findings by showing which associations remain after accounting for diagnosis and which appear to be diagnosis-specific. Clinically, this supports the need for adherence interventions that are not generic, but adapted to diagnostic context and to the specific medication-related concerns of each patient group.

The main contribution of the present study is not simply that medication attitudes and beliefs were associated with adherence, but that these constructs were examined simultaneously in the same analytical framework. This integrated approach shows that adherence cannot be adequately understood through a single psychological construct such as treatment attitude alone. The comparison with Ali et al. is informative in this respect: although that study reported substantially higher DAI-10 scores, it also reported lower MARS scores, indicating that more favorable declared attitudes toward medication do not necessarily translate into better adherence behavior across samples or clinical contexts. This discrepancy supports the need to assess adherence behavior, treatment attitudes, and medication beliefs together, rather than assuming a direct one-to-one relationship between positive attitude and adherence.

A clinically relevant finding was that BMQ-General remained negatively associated with adherence, even when DAI-10 and BMQ-Specific were included in the same model. This suggests that broad beliefs about medicines as a class, including concerns about harm, overuse, or general skepticism toward pharmacological treatment, may be relevant beyond patients’ beliefs about their own prescribed medication. In practical terms, a patient may accept the necessity or usefulness of a specific psychiatric medication while still holding broader negative views about medicines in general. Therefore, interventions that focus only on explaining the benefits of the current prescription may be insufficient if they do not also address general medication skepticism, mistrust of pharmacological treatment, or concerns about long-term harm.

From a clinical perspective, these findings support the potential use of brief assessment of general medication beliefs as part of adherence-risk screening in psychiatric care. BMQ-General may help identify patients whose adherence difficulties are related not only to doubts about a specific medication, but also to broader negative beliefs about medicines. However, the model explained 30.8% of the variance in adherence after adjustment, meaning that approximately 70% of adherence variability remained unexplained. Therefore, belief- and attitude-focused interventions may be clinically useful, but they should not be expected to fully account for adherence behavior on their own. Future adherence interventions should combine belief-focused psychoeducation with assessment of clinical severity, medication class, side effects, illness insight, cognitive functioning, therapeutic alliance, social support, and practical barriers to medication-taking.

An interesting and clinically relevant finding was that female participants reported significantly higher medication adherence despite having lower DAI-10 scores than male participants. At first glance, this appears counterintuitive, because a more positive attitude toward medication would usually be expected to correspond to better adherence. However, this discrepancy suggests that adherence behavior may not depend only on a globally favorable attitude toward treatment. Female participants also reported stronger BMQ-General and BMQ-Specific beliefs, indicating greater cognitive engagement with medication-related issues. Therefore, women may have been more aware of the risks, concerns, and personal implications of psychiatric treatment, but this did not necessarily translate into poorer adherence. Instead, stronger beliefs and concerns may coexist with more consistent medication-taking behavior, possibly because adherence is influenced not only by positive attitudes, but also by perceived responsibility, illness awareness, fear of relapse, therapeutic alliance, family involvement, or stronger engagement with healthcare recommendations.

From a clinical perspective, this finding highlights the importance of distinguishing between medication attitude and medication adherence. A lower DAI-10 score among women should not automatically be interpreted as treatment rejection or non-adherence. It may instead reflect greater ambivalence, concern, or critical reflection about treatment. Conversely, higher DAI-10 scores among men may indicate more favorable declared attitudes, but not necessarily better adherence behavior. Clinicians should therefore assess both attitudinal and behavioral aspects of adherence. For female patients, interventions may need to address medication-related concerns while reinforcing already present adherence behaviors. For male patients, psychoeducation and follow-up should not rely solely on apparently positive attitudes, but should also explore actual medication-taking practices, barriers to routine adherence, and possible discrepancies between stated acceptance of treatment and daily behavior.

This study has several limitations. First, its cross-sectional design does not allow causal conclusions regarding the relationship between medication attitudes, beliefs, and adherence. Second, medication adherence was assessed using a self-report instrument, which may be influenced by recall bias, social desirability, or patients’ subjective interpretation of medication-taking behavior. Future studies should consider combining self-report scales such as MARS-10 with more direct or objective adherence measures, including pharmacy refill records, pill counts, clinician-rated adherence, serum drug levels where clinically appropriate, or electronic medication monitoring systems. Electronic monitoring may be particularly useful because it can provide time-stamped information about medication-taking patterns and may help distinguish consistent adherence from irregular or intermittent use. However, such methods also have limitations, including cost, feasibility in routine psychiatric settings, and the fact that opening a medication container does not necessarily confirm ingestion. Third, BMQ-General and BMQ-Specific were analyzed as total scores, although the BMQ is often interpreted through distinct subscales such as Necessity, Concerns, Harm, and Overuse. Fourth, several clinically important covariates that are well known to influence psychiatric medication adherence were not included in the present model. These include medication class, number of prescribed psychotropic medications, duration of illness, severity of psychiatric symptoms, history and number of previous hospitalizations, treatment duration, therapeutic alliance, insight into illness, and adverse effects or perceived side-effect burden. The absence of these variables limits the ability to determine whether the observed associations between attitudes, beliefs, and adherence remain significant after controlling for clinical severity and treatment-related characteristics. Future studies should include these covariates in multivariable models to provide a more comprehensive explanation of adherence behavior in psychiatric populations. Finally, the generalizability of the findings may be limited by the characteristics of the sample and study setting.

## 5. Conclusions

This study examined medication adherence, attitudes toward treatment, and beliefs about medicines in a psychiatric sample using the MARS-10, DAI-10, BMQ-General, and BMQ-Specific instruments. The findings showed that medication adherence was moderate overall, while attitudes toward medication were generally positive. However, a relevant proportion of participants reported neutral or negative attitudes, indicating that favorable treatment attitudes were not universal across the sample.

The results also showed significant associations between adherence, medication attitudes, and medication-related beliefs. Higher DAI-10 scores were associated with higher MARS-10 scores, whereas higher BMQ-General scores were associated with lower MARS-10 scores. In the regression model, DAI-10, BMQ-General, and BMQ-Specific scores together explained 30.8% of variance after adjustment in MARS-10 adherence scores. These findings suggest that patients’ attitudes toward psychiatric medication and their broader beliefs about medicines are meaningfully related to self-reported adherence behavior.

Because of the cross-sectional design, these findings should be interpreted as associations rather than evidence of causal effects. Therefore, the results do not demonstrate that medication beliefs directly determine adherence, but they indicate that beliefs and attitudes may represent clinically relevant correlates of adherence. In routine psychiatric care, assessing both medication-taking behavior and patients’ beliefs about treatment may help clinicians identify patients who could benefit from additional discussion, psychoeducation, or adherence-focused support. Future longitudinal studies should examine whether changes in medication attitudes and beliefs are followed by changes in adherence over time.

## Figures and Tables

**Table 1:** Distribution of participants according to medication adherence level and attitude toward medication.

Variable	Category	Operational Definition	*n*	%
MARS-10 adherence level	Low adherence	0–4 points	76	25.33
MARS-10 adherence level	Moderate adherence	5–7 points	105	35.00
MARS-10 adherence level	High adherence	8–10 points	119	39.67
DAI-10 attitude type	Negative attitude	<0 points	22	7.33
DAI-10 attitude type	Neutral attitude	0 points	60	20.00
DAI-10 attitude type	Positive attitude	>0 points	218	72.67

Note: Percentages were calculated based on the final analyzed sample of 300 participants. Negative attitude: DAI-10 < 0; neutral attitude: DAI-10 = 0; positive attitude: DAI-10 > 0.

**Table 2:** Gender differences in DAI-10, MARS-10, BMQ-General, and BMQ-Specific scores, including effect sizes and *t*-Test Results.

Scale	Men, M ± SD	Women, M ± SD	Mean Difference, M − F	95% CI for Mean Difference	Cohen’s d	95% CI for d	t	df	*p*- Value	Interpretation
DAI-10	4.20 ± 3.81	2.95 ± 2.92	1.25	0.48 to 2.02	0.370	0.141 to 0.598	3.200	279.239	0.0015	Small-to-moderate; men scored higher
MARS-10	5.93 ± 1.75	6.61 ± 2.61	−0.68	−1.18 to −0.18	−0.306	−0.534 to −0.078	−2.653	260.190	0.0085	Small; women scored higher
BMQ-General	20.54 ± 4.46	22.86 ± 6.74	−2.32	−3.62 to −1.02	−0.406	−0.635 to −0.177	−3.518	258.473	0.0005	Small-to-moderate; women scored higher
BMQ-Specific	30.73 ± 7.18	32.55 ± 4.75	−1.82	−3.20 to −0.44	−0.299	−0.526 to −0.071	−2.589	258.247	0.0102	Small; women scored higher

**Table 3:** Regression with gender added as a covariate.

**Predictor**	**B**	**95% CI**	***p***
DAI-10	0.273	0.212 to 0.333	<0.001
BMQ-General	−0.165	−0.206 to −0.124	<0.001
BMQ-Specific	0.031	−0.007 to 0.069	0.112
Female gender	1.349	0.938 to 1.760	<0.001

**Table 4:** Gender-stratified regressions.

Gender	Predictor	B	95% CI	*p*
Men	DAI-10	0.086	0.010 to 0.162	0.027
Men	BMQ-General	−0.004	−0.071 to 0.064	0.918
Men	BMQ-Specific	0.060	0.016 to 0.103	0.008
Women	DAI-10	0.434	0.327 to 0.541	<0.001
Women	BMQ-General	−0.180	−0.228 to −0.132	<0.001
Women	BMQ-Specific	0.013	−0.057 to 0.083	0.721

**Table 5:** Diagnosis-group descriptive results.

Scale	Depression, n = 160	Schizophrenia, n = 140	Mean Difference	95% CI	*p*	Cohen’s d
DAI-10	4.41 ± 3.71	2.61 ± 2.83	1.80	1.05 to 2.54	<0.001	0.54
MARS-10	6.39 ± 2.28	6.12 ± 2.20	0.27	−0.24 to 0.78	0.294	0.12
BMQ-General	21.30 ± 5.22	22.16 ± 6.43	−0.86	−2.20 to 0.49	0.210	−0.15
BMQ-Specific	34.56 ± 5.24	28.30 ± 5.37	6.26	5.05 to 7.47	<0.001	1.18

**Table 6:** Regression with diagnosis as covariate.

Predictor	B	95% CI	*p*
DAI-10	0.265	0.202 to 0.329	<0.001
BMQ-General	−0.184	−0.230 to −0.137	<0.001
BMQ-Specific	0.096	0.045 to 0.147	<0.001
Diagnosis: schizophrenia	0.961	0.407 to 1.516	0.001

Note: Coding: schizophrenia = 1, depression = 0.

**Table 7:** Diagnosis-stratified regressions.

Diagnosis	Predictor	B	95% CI	*p*
Depression	DAI-10	0.161	0.098 to 0.223	<0.001
Depression	BMQ-General	−0.335	−0.398 to −0.272	<0.001
Depression	BMQ-Specific	0.042	−0.020 to 0.104	0.187
Schizophrenia	DAI-10	0.652	0.561 to 0.743	<0.001
Schizophrenia	BMQ-General	−0.137	−0.179 to −0.094	<0.001
Schizophrenia	BMQ-Specific	0.344	0.287 to 0.401	<0.001

**Table 8:** Model fit.

Diagnosis	R^2^	Adjusted R^2^	Model Test
Depression	0.641	0.634	F(3, 156) = 92.99, *p* < 0.001
Schizophrenia	0.639	0.631	F(3, 136) = 80.25, *p* < 0.001

**Table 9:** Pearson correlations between scales and Bonferroni-corrected correlations.

Correlation	Pearson r	*p*-Value	Bonferroni-Adjusted *p*	Interpretation
DAI-10 vs. MARS	0.447	*p* < 0.001	<0.001	Moderate positive, significant
DAI-10 vs. BMQ-General	−0.191	*p* = 0.0009	0.0053	Weak negative, significant
DAI-10 vs. BMQ-Specific	0.016	*p* = 0.7778	1.000	No significant correlation
MARS vs. BMQ-General	−0.406	*p* < 0.001	<0.001	Moderate negative, significant
MARS vs. BMQ-Specific	−0.081	*p* = 0.1601	0.970	No significant correlation
BMQ-General vs. BMQ-Specific	0.507	*p* < 0.001	<0.001	Moderate positive, significant

**Table 10:** Model summary.

Indicator	Value
R	0.562
R^2^	0.315
Adjusted R^2^	0.308
F(3, 296)	45.459
*p*-value	<0.001

**Table 11:** Regression coefficients.

Predictor	B	Std. Error	β	t	*p*-Value
Intercept	7.396	0.599	—	12.350	<0.001
DAI-10	0.241	0.032	0.371	7.494	<0.001
BMQ-General	−0.151	0.022	−0.391	−6.821	<0.001
BMQ-Specific	0.040	0.021	0.111	1.968	0.050

**Table 12:** Regression diagnostic statistics for the prediction of MARS scores.

Diagnostic	Result	Interpretation
DAI-10 tolerance/VIF	0.946/1.057	No multicollinearity
BMQ-General tolerance/VIF	0.703/1.422	No multicollinearity
BMQ-Specific tolerance/VIF	0.730/1.370	No multicollinearity
Shapiro–Wilk test of residuals	W = 0.947, *p* < 0.001	Residuals departed from strict normality
Residual skewness	−0.070	No relevant skewness
Residual excess kurtosis	−1.267	Platykurtic residual distribution
Durbin–Watson statistic	2.099	No evidence of autocorrelation
Breusch–Pagan test	LM = 14.771, *p* = 0.002	Evidence of heteroscedasticity
Maximum standardized residual	2.20	No severe outliers
Maximum Cook’s distance	0.030	No influential cases
Maximum leverage	0.051	Some leverage, but not influential

## Data Availability

The original contributions presented in this study are included in the article. Further inquiries can be directed to the corresponding author.
